# Mitochondrial fission-induced mtDNA stress promotes tumor-associated macrophage infiltration and HCC progression

**DOI:** 10.1038/s41388-019-0772-z

**Published:** 2019-03-20

**Authors:** Dengke Bao, Jing Zhao, Xingchun Zhou, Qi Yang, Yibing Chen, Jianjun Zhu, Peng Yuan, Jin Yang, Tao Qin, Shaogui Wan, Jinliang Xing

**Affiliations:** 1 Center for Molecular Pathology, First Affiliated Hospital, Gannan Medical University, Ganzhou, Jiangxi 341000 China; 2 State Key Laboratory of Cancer Biology and Experimental Teaching Center of Basic Medicine, Fourth Military Medical University, Xi’an, Shanxi 710032 China; 3 Department of Hepatobiliary Pancreatic Surgery, Henan Provincial People’s Hospital, Henan University, Zhengzhou, Henan 450002 China; 4 Genetic and Prenatal Diagnosis Center, Department of Gynecology and Obstetrics, First Affiliated Hospital, Zhengzhou University, Zhengzhou, 450052 Henan China; 5 Institue of Preventive Genomic Medicine, School of Life Sciences, Northwest University, Xi’an, 710069 China

**Keywords:** Liver cancer, Immunosurveillance

## Abstract

Tumor-associated macrophages (TAMs) contribute to hepatocellular carcinoma (HCC) progression. However, the molecular mechanism underlying the infiltration of TAMs into HCC microenvironment is largely unclear. Recent studies have reported that alteration of mitochondrial nucleoid structures induces mitochondrial DNA (mtDNA) release into the cytosol, which is recognized as mtDNA stress, and consequently regulates innate immunity. Here we aimed to investigate whether mitochondrial fission induces mtDNA stress and then promotes TAM infiltration and HCC progression. Confocal microscopy and real-time PCR were used to detect cytosolic mtDNA content in HCC cells. The relationship between the expression of mitochondrial fission key regulator dynamin-related protein 1 (Drp1) and the percentage of CD163 (a marker of TAMs)-positive cells was investigated in HCC tissues using immunohistochemistry. Finally, the effect of Drp1 overexpression in HCC cells on recruitment and polarization of TAMs was investigated. Our data showed that increased Drp1 expression was positively correlated with the infiltration of TAMs into HCC tissues. Drp1-mediated mitochondrial fission induced the cytosolic mtDNA stress to enhance the CCL2 secretion from HCC cells by TLR9-mediated NF-κB signaling pathway, and thus promoted the TAM recruitment and polarization. Depleting cytosolic mtDNA using DNase I or blocking TLR9 pathway by TLR9 antagonist, siRNA for TLR9 or p65 in HCC cells with Drp1 overexpression significantly decreased the recruitment and polarization of TAMs. Blocking CCR2 by antagonist significantly reduced TAM infiltration and suppressed HCC progression in mouse model. In conclusion, our findings reveal a novel mechanism of TAM infiltration in HCC by mitochondrial fission-induced mtDNA stress.

## Introduction

Hepatocellular carcinoma (HCC) is one of the most common cancer and a leading cause of cancer-related deaths worldwide [1]. Previous studies have shown that the interaction between HCC cells and tumor microenvironment plays an important role in HCC progression. Revealing the underlining mechanism of interaction between HCC cells and tumor microenvironment components may be useful for the discovery of novel therapeutic targets [2]. Tumor-associated macrophages (TAMs) are the most abundant component residing in the tumor microenvironment [2, 3]. TAMs originate from myelomonocytic cells, and are recruited to the tumor microenvironment by tumor-derived cytokines and chemokines, including chemokine (C–C motif) ligand 2 (CCL2), vascular endothelial growth factor (VEGF), macrophage colony-stimulating factor (M-CSF), and transforming growth factor beta (TGF-β) [4, 5]. Within the tumor microenvironment, TAMs are identified as alternatively activated (M2) macrophages, which are characterized by poor capability to present antigen, distinctive expression of cytokines and chemokines, such as interleukin (IL)-10, CCL17, CCL22, and TGF-β [3–5]. Previous studies have indicated that TAMs promote angiogenesis, metastasis, and immune suppression in cancers through the secretion of cytokines, chemokines, growth factors, and matrix metalloproteases. Indeed, increased TAM infiltration is associated with poor prognosis in HCC [2, 4, 5]. Previous studies have demonstrated that nuclear factor-κB (NF-κB), STAT-3, and hypoxia-inducibe factor-1 signaling pathways are involved in TAM recruitment and polarization [4]. However, the mechanism underlying the effect of HCC cells on TAM infiltration is not well known.

The vital roles of mitochondria in immunity response have been well documented. Previous studies have demonstrated that mitochondria play multifunctional roles in various malignant tumor progression by modulating cell cycle, gene expression, metabolism, cell viability, and other aspects of cell growth and stress responses [6, 7]. Recently, cumulative evidence reveals that abnormal mitochondrial fission is implicated in the pathogenesis of many malignancies [6, 7]. Our previous studies have demonstrated that dynamin-related protein 1 (Drp1), the most important protein for mitochondrial fission, plays a critical role in HCC progression by regulation of cell survival and metastasis [8, 9]. The dynamic change of mitochondrial fusion and fission is critical for mitochondrial DNA (mtDNA) distribution and mitochondrial homeostasis [10–12]. Previous studies have shown that mitochondrial dynamics play an important role in mtDNA nucleoid distribution, cristae reformation, and the proapoptotic status of mitochondria [10, 11]. Transcription factor A, mitochondrial (TFAM) deficiency markedly alters mtDNA packaging, organization and distribution of nucleoids, and induces the release of mtDNA into cytosol, which is recognized as cytosolic mtDNA stress [13]. However, whether mitochondrial fission induces mtDNA stress and is involved in HCC progression is worthy to be further investigated.

In this study, we examined the effect of Drp1-mediated mitochondria fission on release of mtDNA into cytosol and explored whether the cytosolic mtDNA stress is involved in polarization and recruitment of TAMs by in vitro cell co-culture assay and in vivo animal model. Our findings provide new insight into the molecular mechanisms underlying the crosstalk between HCC cells and TAMs.

## Results

### Increased mitochondrial fission positively correlated with TAM infiltration in HCC tissues

**Fig. 1:**
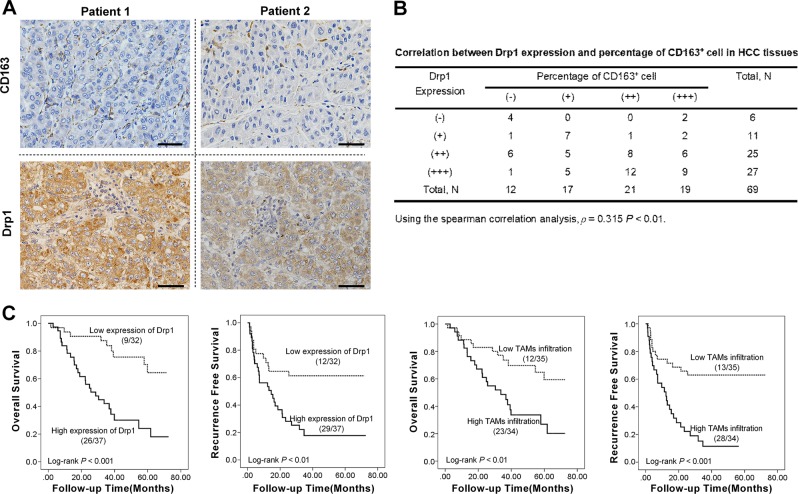
Increased mitochondrial fission positively correlated with tumor-associated macrophage (TAM) infiltration in hepatocellular carcinoma (HCC) tissues. **a** Representative immunohistochemical (IHC) staining images of dynamin-related protein 1 (Drp1) and CD163 in representative HCC tissues (*n* = 69). Scale bar: 50 μm. **b** Correlation between Drp1 expression and percentage of CD163^+^ cells in HCC tissues. Patients were divided into four groups by the quartile value of Drp1 expression or percentage of CD163^+^ cells for further analysis. **c** Kaplan-Meier curve analysis of overall survival and recurrence-free survival in HCC patients by the expression of Drp1 and TAM infiltration in HCC tissues. Patients were divided into high or low level by the median value of Drp1 expression or percentage of CD163^+^ cells for further analysis. TAM infiltration was defined by the percentage of CD163^+^ cells. Death/total and recurrence/total number of patients in each subgroup were presented

Drp1 is one of the most important mitochondrial fission regulator, which can be used as an indirect marker to indicate the status of mitochondrial fission [14]. A series of previous studies have demonstrated the link between mitochondrial fission and the expression of Drp1 in various tumor cells [15, 16]. Our previous study has also reported that mitochondrial fission level examined by electron microscope is significantly correlated with Drp1 expression in HCC tissues [8]. To further investigate the relationship between mitochondrial fission and TAM infiltration, the expression of Drp1 and CD163 (a marker of TAMs) was indirectly examined in 69 paired tumor and peritumor tissues from HCC patients using immunohistochemistry (IHC) and the percentage of CD163-positive cells was counted (Fig. 1a). Our data clearly confirmed the upregulated expression of Drp1 and the significant infiltration of CD163-positive cells in HCC tissues. Importantly, we found that the expression of Drp1 was significantly positively correlated with the percentage of CD163-positive cells in HCC tissues (*ρ* = 0.315, *p* < 0.01), suggesting a clear link between increased mitochondrial fission and TAM infiltration (Fig. 1b). In addition, our results also showed that HCC patients with high Drp1 expression or high TAM infiltration had a significantly poorer overall survival (log rank *p* < 0.001 and *p* < 0.01, respectively) and recurrence-free survival (log rank *p* < 0.01 and *p* < 0.001, respectively) than those with low Drp1 expression or low TAM infiltration, respectively (Fig. 1c).

### Increased mitochondrial fission promoted the secretion of CCL2 from HCC cells

We first established HCC cell models with different levels of mitochondrial fission by overexpression or knockdown of Drp1, which was validated at mRNA and protein levels by quantitative real-time PCR (qPCR) and western blot, respectively (Supplementary Figure. 1A, 1C, 1E). Furthermore, we confirmed that Drp1 overexpression significantly increased the mitochondrial fission in both SNU-739 and MHCC97L cells (Supplementary Figure. 1B and 1D). In contrast, Drp1 knockdown remarkably reduced the percentage of fragmented mitochondria in SNU-739 cells (Supplementary Figure. 1F). These results are consistently with our previous study [8].

**Fig. 2:**
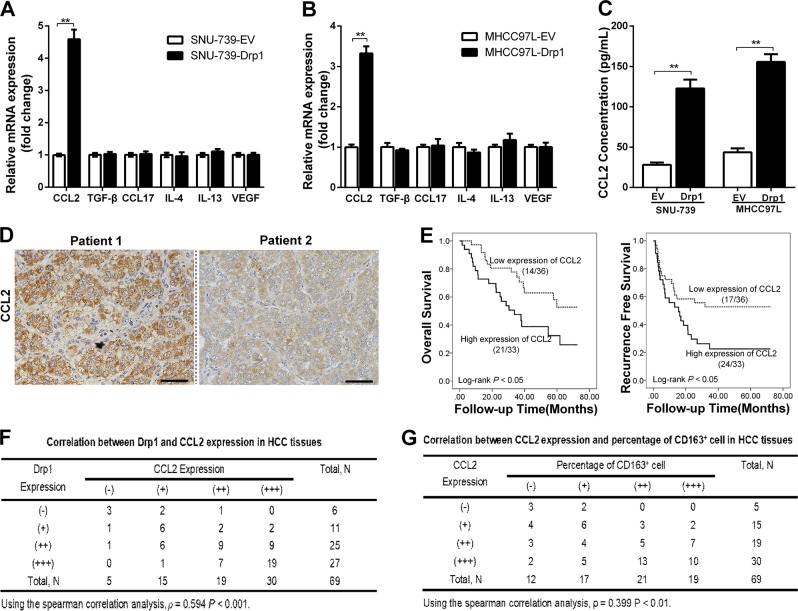
Increased mitochondrial fission promoted the secretion of chemokine (C–C motif) ligand 2 (CCL2) from hepatocellular carcinoma (HCC) cells. **a**, **b** Quantitative real time polymerase chain reaction (qRT-PCR) analysis for the mRNA expression levels of genes coding for tumor-associated macrophages (TAMs) recruitment associated cytokines in HCC cells with dynamin-related protein 1 (Drp1) overexpression. **c** Enzyme-linked immunosorbent assay analysis of CCL2 concentration in the supernatants of cultured HCC cells with Drp1 overexpression. **d** Representative immunohistochemical staining images of CCL2 in representative HCC tissues (*n* = 69). Scale bar: 50 μm. **e** Kaplan-Meier curve analysis of overall survival and recurrence-free survival in HCC patients by the expression of CCL2 in HCC tissues. Patients were divided into high or low level by the median value of CCL2 expression for further analysis. Death/total and recurrence/total number of patients in each subgroup were presented. **f** Correlation between CCL2 and Drp1 expression in HCC tissues. **g** Correlation between CCL2 expression and percentage of CD163^+^ cell in HCC tissues. Patients were divided into four groups by the quartile value of Drp1, CCL2 expression, or percentage of CD163^+^ cells for further analysis. EV indicated as cells transfected with empty vector; Drp1 indicated as cells transfected with vector expressing Drp1. Data shown are the mean ± s.e.m. from three independent experiments. **p < 0.01

To explore the potential effect of mitochondrial fission on TAM infiltration and underlying mechanisms, we first examined the expression and secretion of major cytokines and chemokines, including CCL2, CCL17, TGF-β, IL-4, IL-13, and VEGF, which play important roles in regulation of TAM infiltration, in HCC cells with Drp1 overexpression or knockdown using quantitative PCR and enzyme-linked immunosorbent assay (ELISA). As shown in Fig. 2a–c, Drp1 overexpression significantly increased the mRNA expression and protein secretion of CCL2 in both SNU-739 and MHCC97L HCC cells. In contrast, mRNA expression and protein secretion levels of CCL2 were not significantly affected by Drp1 knockdown (Supplementary Figure. 1G). Moreover, our IHC data indicated that HCC patients with high CCL2 expression had a significantly poorer overall survival (log rank *p* < 0.05) and recurrence-free survival (log rank *p* < 0.05) than those with low CCL2 expression (Fig. 2d, e). As expected, CCL2 expression was significantly correlated with Drp1 expression (*ρ* = 0.594, *p* < 0.001) and the percentage of CD163-positive cells in HCC tissues (*ρ* = 0.399, *p* < 0.01) (Fig. 2f, g). These findings indicated that increased mitochondrial fission promoted the secretion of CCL2 from HCC cells, which was correlated with TAM infiltration in HCC tissues.

### Increased mitochondrial fission induced cytosolic mtDNA stress in HCC cells

**Fig. 3:**
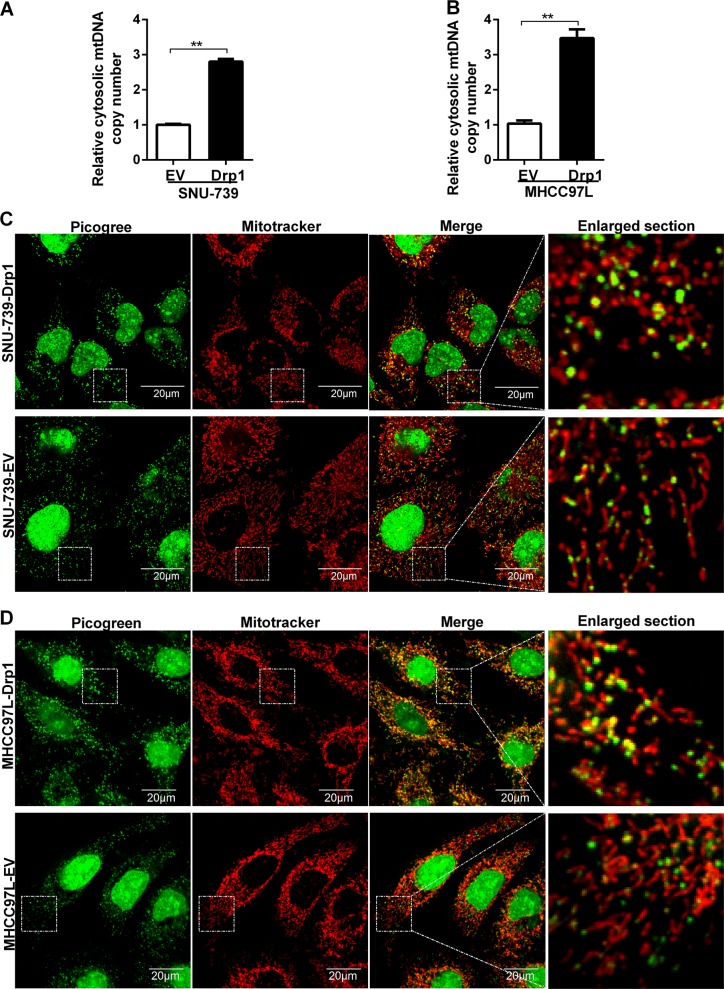
Increased mitochondrial fission induced cytosolic mitochondrial DNA (mtDNA) stress in hepatocellular carcinoma (HCC) cells. **a**, **b** Cytosolic mtDNA copy number was quantitated via quantitative real-time PCR in HCC cells as indicated. HGB1 was used as internal control. **c**, **d** Confocal microscopy images of indicated HCC cells stained with Picogreen (DNA) and MitoTracker (Mito.). EV indicated as cells transfected with empty vector; Drp1 indicated as cells transfected with vector expressing dynamin-related protein 1. Data shown are the mean ± s.e.m. from three independent experiments. **p < 0.01

To further elucidate the mechanism underlying mitochondrial fission-mediated CCL2 secretion, we investigated whether increased mitochondrial fission could induce mtDNA stress, which is characterized as release of mtDNA into the cytosol in HCC cells. As shown in Fig. 3a, b, Drp1 overexpression significantly increased cytosolic mtDNA copy number in both SNU-739 and MHCC97L HCC cells. Moreover, when compared with control cells, HCC cells with Drp1 overexpression exhibited a larger size distribution of mitochondrial nucleoids, indicating the altered packaging and organization of mtDNA (Fig. 3c, d). In contrast, cytosolic mtDNA copy number and mitochondrial nucleoids were not significantly affected by Drp1 knockdown (Supplementary Figure. 2A and 2B). Our data indicate that increased mitochondrial fission induces the release of mtDNA into the cytosol, which causes the cytosolic mtDNA stress.

### Cytosolic mtDNA stress promoted the secretion of CCL2 by TLR9-mediated NF-κB signaling pathway

**Fig. 4:**
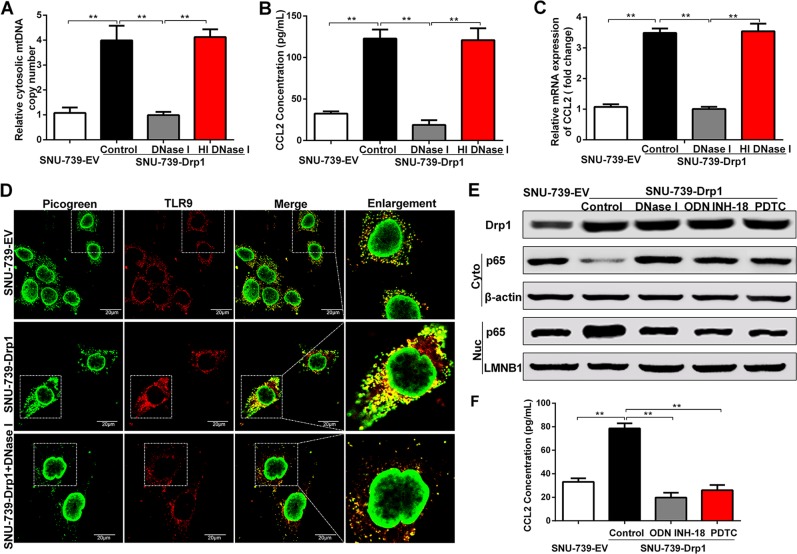
Cytosolic mitochondrial DNA (mtDNA) stress promoted the secretion of chemokine (C–C motif) ligand 2 (CCL2) by TLR9-mediated nuclear facor-κB (NF-κB) signaling pathway. **a** Cytosolic mtDNA copy number was quantitated via quantitative real-time PCR in hepatocellular carcinoma (HCC) cells as indicated. HGB1 was used as internal control. **b** Enzyme-linked immunosorbent assay (ELISA) analysis of the CCL2 concentration in the supernatants of cultured HCC cells as indicated. **c** Quantitative real time polymerase chain reaction (qRT-PCR) analysis for the mRNA expression levels of CCL2 in HCC cells treated as indicated. **d** Co-localization analyses between mtDNA (Picogreen) and TLR9 (PE-label antibody for TLR9) in indicated HCC cells by confocal microscopy. **e** Western blot analyses for protein levels of dynamin-related protein 1 (Drp1) and NF-κB activation-related molecules in whole cells or p65 in cytoplasm and nucleus of HCC cells as indicated. **f** ELISA analysis of the CCL2 concentration in the supernatants of cultured HCC cells with treatment as indicated. EV indicated as cells transfected with empty vector, Drp1 indicated as cells transfected with vector expressing Drp1. DNase I, HCC cells treated with DNase I preparations; HI DNase I, HCC cells treated with heat-inactivated DNase I preparations; ODN INH-18, HCC cells treated with TLR9 antagonist (ODN INH-18); PDTC, HCC cells treated with NF-κB inhibitor PDTC; p65, NF-κB p65; p-p65, phosphorylated NF-κB p65; Control, SNU-739-Drp1 HCC cells without treatment. Data shown are the mean ± s.e.m. from three independent experiments. **p < 0.01

Previous studies have well documented that mtDNA is one major type of damage-associated molecular patterns (DAMPs), which can activate the DNA sensor TLR9 signaling to trigger the inflammatory responses [17–19]. Therefore, we explored the functional roles of cytosolic mtDNA stress in mitochondrial fission-induced secretion of CCL2 and underlying molecular mechanism. As shown in Fig. 4a–c, we found that depleting cytosolic mtDNA by DNase I treatment significantly reduced the Drp1 overexpression-induced secretion and mRNA expression of CCL2 in HCC cells. However, treatment with control protein or heat-inactivated DNase I did not affect cytosolic mtDNA copy number and the CCL2 secretion and mRNA expression in HCC cells. These findings indicate that mitochondrial fission-induced secretion of CCL2 is mediated by cytosolic mtDNA stress.

Furthermore, we explored whether activation of TLR9-mediated signaling pathways was involved in cytosolic mtDNA stress-mediated CCL2 secretion. Immunofluorescent staining assay showed that HCC cells with Drp1 overexpression exhibited a significantly increased co-localization of mtDNA and TLR9 than control cells, and DNase I treatment remarkably reversed the effect of Drp1 overexpression (Fig. 4d). We next sought to investigate whether mtDNA and TLR9 co-localization mediates the activation of NF-κB signaling pathway, which is a common downstream of TLR9 [20]. Our data showed that the nuclear translocation of phosphorylated NF-κB p65 was significantly increased by Drp1 overexpression, which could be remarkably inhibited by treatment with DNase I, TLR9 antagonist (ODN INH-18), or NF-κB inhibitor PDTC (Fig. 4e). Similarly, knockdown of p65 or TLR9 expression by small interfering RNA (siRNA) significantly abolished the nuclear translocation of phosphorylated NF-κB p65 induced by Drp1 overexpression (Supplementary Figure. 3A and 3B). Drp1-mediated CCL2 secretion was also significantly inhibited by treatment with ODN INH-18, PDTC (Fig. 4f), or siRNA for p65 and TLR9 (Supplementary Figure. 3C and 3D). In addition, HCC patients with high TLR9 expression had a significantly poorer overall survival (log rank *p* < 0.01) and recurrence-free survival (log rank *p* < 0.01) than those with low TLR9 expression (Supplementary Figure. 4A and 4B). Collectively, these data suggest that cytosolic mtDNA stress may promote the secretion of CCL2 through TLR9-mediated NF-κB signaling pathway, which plays a critical role in prognosis of HCC patients.

In addition, we investigated the reactive oxygen species (ROS) levels in HCC cells. As shown in Supplementary Figure. 5A-5C, our study further indicated that the increased mitochondrial fission induced the elevation of ROS levels, which was markedly reversed by MitoTEMPO, a specific scavenger of mitochondrial superoxide. However, the ROS elevation was significantly reduced by treatment with DNase I, TLR9 antagonist, or TLR9 siRNA, indicating that the mtDNA stress-mediated activation of TLR9 signaling was very important for ROS production and NF-κB activation.

### Mitochondrial fission-induced cytosolic mtDNA stress promoted macrophage recruitment and polarization by CCL2 in HCC

**Fig. 5:**
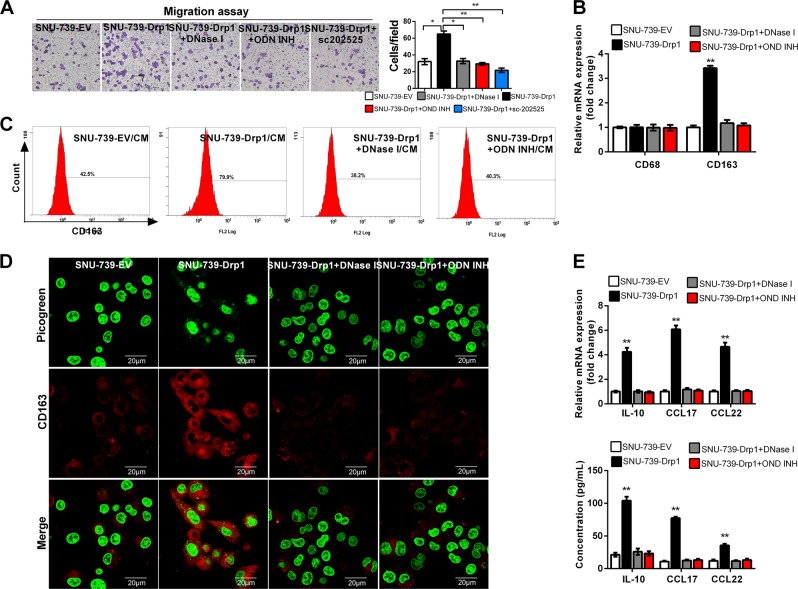
Mitochondrial fission-induced cytosolic mitochondrial DNA (mtDNA) stress promoted macrophage recruitment and polarization in hepatocellular carcinoma (HCC) by chemokine (C–C motif) ligand 2 (CCL2). **a** Transwell migration assay of macrophage by CM from HCC cells as indicated. **b** Quantitative real time polymerase chain reaction (qRT-PCR) analysis for the expression levels of CD68 and CD163 in THP-1 macrophages treated with CM from HCC cells as indicated. **c**, **d** Flow cytometry and immunofluorescence analysis for the expression levels of CD163 in THP-1 macrophages treated with CM from HCC cells as indicated. **e** Quantitative real time polymerase chain reaction (qRT-PCR) and enzyme-linked immunosorbent assay for the mRNA expression and secretion of tumor-associated macrophage (TAM) characteristic cytokines in THP-1 macrophages treated with CM from HCC cells as indicated. CM conditioned medium. EV indicated as cells transfected with empty vector; Drp1 indicated as cells transfected with vector expressing dynamin-related protein 1. DNase I, HCC cells treated with DNase I preparations; ODN INH-18, HCC cells treated with TLR9 antagonist (ODN INH-18); sc-202525, macrophages treated with a CCR2 antagonist. Data shown are the mean ± s.e.m. from three independent experiments. *p < 0.05; **p < 0.01

To test whether increased CCL2 secretion mediated by mitochondrial fission-induced cytosolic mtDNA stress plays an important role in macrophage infiltration, we first examined the effect of conditioned media (CM) from HCC cells with increased mitochondrial fission on macrophage recruitment ability by in vitro migration assay. As shown in Fig. 5a, our data showed that CM from Drp1-overexpressed HCC cells significantly increased the recruitment of macrophages when compared with CM from control cells. However, treatment with DNase I or ODN INH-18 in Drp1-overexpressed cells abolished this effect. Furthermore, treatment with a CCR2 antagonist sc-202525 in macrophages also significantly inhibited the recruitment effect. Together, these results suggest that mitochondrial fission-induced cytosolic mtDNA stress may promote the recruitment of macrophages by secretion of CCL2.

Next, we investigated whether mitochondrial fission-induced cytosolic mtDNA stress promotes the polarization of macrophages by CCL2. As shown in Fig. 5b, macrophages treated with CM from Drp1-overexpressed HCC cells exhibited a markedly increased mRNA expression of TAM marker CD163 when compared with corresponding control group. As expected, treatment with DNase I or ODN INH-18 in HCC cells abolished this Drp1 overexpression-mediated effect. In comparison, the marker of macrophage CD68 was not affected. Similar results were obtained at protein level of CD163 by flow cytometry and immunofluorescence assay (Fig. 5c, d). Furthermore, the mRNA expression and protein secretion of TAM characteristic cytokines IL-10, CCL17, and CCL22 were significantly increased in macrophages incubated with CM from HCC cells with Drp1 overexpression when compared with control group (Fig. 5e). However, treatment with DNase I or ODN INH-18 in HCC cells with Drp1 overexpression abolished this effect. Altogether, these results suggest that mitochondrial fission-induced cytosolic mtDNA stress may promote the recruitment and polarization of macrophages by CCL2 in HCC.

### Mitochondrial fission-induced cytosolic mtDNA stress promoted TAM-mediated HCC progression

**Fig. 6:**
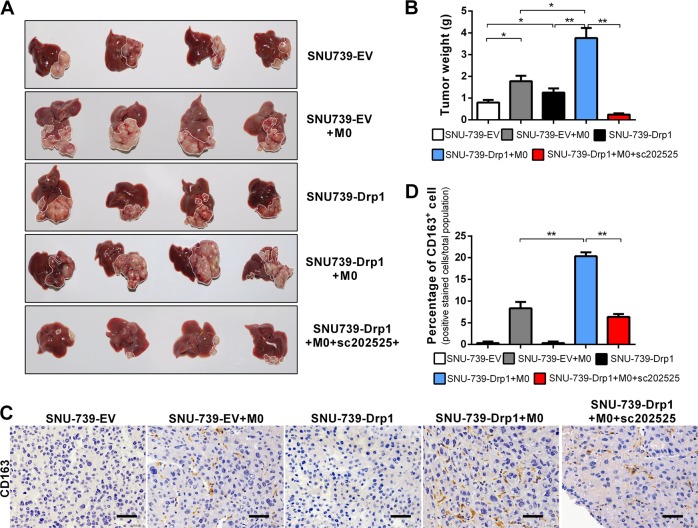
Mitochondrial fission-mediated cytosolic mitochondrial DNA stress promoted tumor-associated macrophage-mediated hepatocellular carcinoma (HCC) progression. **a** Representative images of orthotopic HCC tissues with treatment as indicated. **b** Tumor weight was examined in individual of A. **c** Hematoxylin and eosin staining and immunohistochemistry (CD163) were performed in orthotopic HCC tissues with treatment as indicated. Scale bar: 50 μm. **d** The percentage of CD163-positive macrophage in C was statistical analysis (*n* = 4). EV indicated mice were orthotopically injected with HCC cells stable transfection with empty vector; Drp1 mice were orthotopically injected with HCC cells stable transfection with vector expressing dynamin-related protein 1. M0, mice were injected with macrophages; sc-202525, mice were injected with a CCR2 antagonist. Data shown are the mean ± s.e.m. *p < 0.05; **p < 0.01

Finally, we investigated the effect of mitochondrial fission-induced cytosolic mtDNA stress on TAM-mediated HCC progression in the orthotopic nude mice model of HCC. The nude mice were sacrificed for the tumor growth evaluation 5 weeks after intrahepatic implantation. As shown in Fig. 6a, b, either Drp1 overexpression or injection of THP-1 macrophages significantly increased the weight of orthotopic tumor in comparison to control group. Moreover, the injection of THP-1 macrophages and Drp1 overexpression exhibited a significantly combination effect on tumor growth, which can be remarkably attenuated by sc-202525 treatment. We further investigated the TAM infiltration in the orthotopic lesions by IHC staining using human CD163 monoclonal antibody (10D6), which is specifically reacted with human samples. As shown in Fig. 6c, d, Drp1 overexpression significantly increased TAM infiltration in tumor microenvironment, which can be abolished by sc-202525. These results clearly confirmed the in vitro findings that mitochondrial fission-mediated cytosolic mtDNA stress promoted TAM infiltration and HCC progression.

## Discussion

**Fig. 7:**
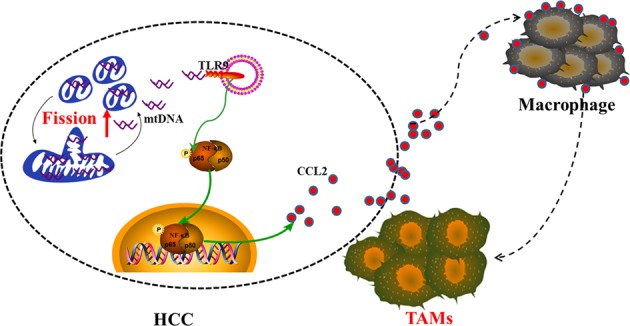
Schematic depicting the effect of increased mitochondrial fission in hepatocellular carcinoma (HCC) cells on tumor-associated macrophage recruitment and polarization and underlying mechanism

Accumulating evidence indicates that the crosstalk between HCC cells and TAMs in the tumor microenvironment plays an important role in HCC progression [2, 3]. The molecular mechanism underlying the interaction between HCC cells and TAMs is not completely known. Here we for the first time demonstrate that increased mitochondrial fission significantly induces cytosolic mtDNA stress in HCC cells and present a critical finding that mitochondrial fission-induced mtDNA stress promotes the TAM infiltration and HCC progression by activating TLR9-mediated NF-κB signaling pathway to increase the production of CCL2 (Fig. 7).

Previous studies have shown that mitochondrial dynamics consisting of fission and fusion plays important roles in regulating the nucleoid structure of mtDNA, cristae reformation, and the proapoptotic status of mitochondria [10, 11]. Consistent with previous findings, we found that HCC cells with Drp1 overexpression exhibited a larger size distribution of mitochondrial nucleoids, indicating the altered packaging and organization of mtDNA. Moreover, previous studies have shown that TFAM deficiency results in the aberrant distribution of nucleoid and thus induces cytosolic mtDNA stress [13]. In the present study, our data demonstrated that increased mitochondrial fission induced release of mtDNA into the cytosol in HCC cells, which is commonly recognized as cytosolic mtDNA stress, suggesting a potentially critical role of mitochondrial fission-regulated mtDNA hemostasis in hepatocarcinogenesis.

To date, detailed mechanisms underlying mitochondrial fission-mediated mtDNA release remain to be explored. A previous study has shown that Drp1-induced mitochondrial fission stimulates hemifusion of liposomes to affect the integrity of mitochondrial outer membrane (OM) [21]. Another study has reported that Drp1-induced mitochondrial fission stimulates the release of Cyt c after the Bax/Bak-dependent release of Smac/DIABLO, but does not directly regulates mitochondrial OM permeabilization (MOMP) [22]. These results suggest that complicated mechanism including MOMP and apoptosis may be implicated in the release of intramitochondrial contents. In addition, previous studies have demonstrated that the mitochondrial fission and fusion machinery coordinates with mitochondrial nucleoid maintenance to ensure the efficient distribution of mtDNA throughout the cell [14]. Consistently, we found the significant nucleoid clustering in HCC cells with increased mitochondrial fission, which provides further support for the functional roles of mitochondrial nucleoid packaging and organization in mtDNA distribution. Certainly, further studies are needed to provide new insights into the molecular mechanisms by which mitochondrial fission regulates the cytosolic release of mtDNA.

As an important component of the tumor microenvironment, TAMs have been reported to be widely implicated in HCC progression. Clinical studies have shown that increased TAM infiltration is frequently correlated with poor HCC prognosis [4, 23]. However, few studies have elucidated the relationship between mitochondrial dynamics in HCC cells and TAM recruitment and polarization in tumor microenvironment. Our study indicated that the expression of Drp1 was significantly positively correlated with the percentage of CD163-positive cells in HCC tissues, suggesting a clear link between increased mitochondrial fission and TAM infiltration.

TAMs are recruited to the tumor microenvironment commonly by tumor-derived cytokines and chemokines, including CCL2, VEGF, M-CSF, and TGF-β [4, 5]. Recent investigations have suggested that HCC cells in hypoxic microenvironment promote TAM recruitment and polarization by secretion of cytokines and thus induce an immunosuppressive tumor microenvironment to promote metastasis [24–26]. Furthermore, hypoxia has been found to upregulate the expression of Drp1 and stimulate mitochondrial fission [27–29]. In our study, we found that increased mitochondrial fission in HCC cells promoted TAM recruitment and polarization, which required cytosolic mtDNA stress. In addition, previous studies have shown that mitochondrial dynamics plays an important role in regulation of immune responses, including production of inflammatory cytokines [30–32]. Consistent with previous findings [5], our study showed that increased mitochondrial fission induced CCL2 secretion, which is the most vital chemoattractant for TAM recruitment and polarization.

CCL2 is a multifunctional factor involved in various aspects of liver disease pathogenesis, including cirrhosis and hepatocarcinogenesis [5, 33]. Previous studies have shown that CCL2 promotes the accumulation and polarization of macrophages by CCR2 receptor signaling pathway [5, 33, 34]. Moreover, a previous study has found that targeting CCL2/CCR2 signaling with a CCR2 antagonist significantly reduces the recruitment and polarization of TAMs and thus enhances tumor immunotherapeutic effect [5]. Consistently, our study demonstrated that CCL2 was a critical mediator to link the cytosolic mtDNA stress in HCC cells and TAM infiltration. We further confirmed this finding in an orthotopic nude mice model of HCC by injection of CCR2 antagonist.

In addition to biosynthesis and bioenergetics, mitochondria are emerging as an important source of endogenous DAMPs, which play crucial roles in the activation of innate immune response and pathological processes [18, 35, 36]. As a major mitochondrial DAMP, mtDNA contains the motifs unmethylated CpG DNA, which can elicit inflammatory responses by activation of TLR9 signaling pathway [17–19]. TLR9 is an endosomal receptor frequently upregulated in many cancer types, including HCC [37]. Consistent with these previous reports, we found that HCC patients with high TLR9 expression had a significantly poorer overall survival and recurrence-free survival. Our study also found that the Drp1 overexpression-mediated CCL2 production and nuclear translocation of phosphorylated p65 were significantly suppressed by treatment with DNase I, TLR9 antagonist, NF-κB inhibitor, or siRNAs for TLR9 and p65. Collectively, these findings indicate that TLR9-mediated NF-κB signaling pathway involved in mtDNA stress-induced the production and secretion of CCL2.

Accumulating evidence has demonstrated that abnormal mitochondrial dynamics is involved in cancer progression [15, 16]. Our previous studies have demonstrated that mitochondrial dynamics plays a critical role in regulation of HCC cell survival by mediating ROS production [8]. Consistent with our previous findings, our study further confirmed that increased mitochondrial fission induced the elevation of ROS levels (Supplementary Figure. 5). However, the ROS elevation and NF-κB activation were significantly suppressed after treatment with DNase I, TLR9 antagonist, or TLR9 siRNA (Fig. 4, Supplementary Figure. 3A, Fig. 5B and 5C). These results indicated that mtDNA stress-mediated activation of TLR9 signaling contributed ROS production and NF-κB activation, which was consistent with previous studies [38, 39]. Furthermore, previous studies have reported that TLR9 knockdown or treatment with TLR9 antagonist significantly reduces ROS production [40, 41]. Collectively with our founding, ROS elevation induced by mitochondrial fission may be mainly due to mtDNA stress. On the other hand, our study also suggested that ROS production may derive from mitochondria, which was markedly reversed by MitoTEMPO, a specific scavenger of mitochondrial superoxide (Supplementary Figure. 5A). Although other mechanisms underlying the ROS production and activation of NF-κB need to be further ruled out, targeting TLR9 could be a potential treatment strategy for HCC patients.

In summary, we observed a cytosolic mtDNA stress in HCC cells with increased mitochondrial fission, which contributed to TAM recruitment and polarization by upregulating CCL2 secretion and consequently promoted HCC progression. The results shed light on the interaction between HCC cells and TAMs mediated by mitochondrial fission-induced mtDNA stress. Furthermore, the present study extends our understanding on the mechanisms underlying critical role of mitochondrial fission in HCC progression.

## Materials and methods

### HCC patients and formalin-fixed paraffin-embedded tissue samples

A total of 69 paired HCC and adjacent non-HCC formalin-fixed paraffin-embedded tissue samples from HCC patients who underwent surgery treatment were collected at Department of Pathology of Xijing hospital affiliated with the Fourth Military Medical University (FMMU) in Xi’an, China. The demographic variables, and clinical and follow-up data of each patient were summarized in Supplementary Table 1. The latest follow-up date was July 2013 and the median follow-up duration was 22.9 (2.3–45) months. This study was approved by the institutional review board of FMMU and the informed consents were signed by all participants.

### Cell culture and reagents

Human monocytic THP-1 cells (TIB-202D; American Type Culture Collection, Manassas, VA, USA) and HCC cell lines SNU-739 were routinely cultured in RPMI-1640 (Invitrogen, Carlsbad, CA) medium supplemented with 10% fetal bovine serum (FBS; Hyclone, Logan, UT). MHCC97L was maintained in Dulbecco’s modified Eage’s medium (Invitrogen, Carlsbad, CA) medium supplemented with 10% FBS. All HCC cell lines were authenticated using short tandem repeat DNA testing by the FMMU Center for DNA Typing in 2016. THP-1 monocytes were differentiated into macrophages (known as THP-1 macrophages) after 24 h incubation with 150 nM phorbol 12-myristate 13-acetate (P1585, Sigma-Aldrich, St Louis, MO).

SNU-739 and MHCC97L cells with stable overexpression of Drp1 and SNU-739 cells with knockdown of Drp1 were established as previously described [9]. To prepare HCC CM, 5 × 10^6^/ml HCC cells with different treatments were washed three times with serum-free RPMI-1640 and kept in fresh serum-free RPMI-1640 for another 72 h. Then, the supernatant was filtered using 0.22 μm filters and collected as CM, stored at 4 °C for further studies.

The antibodies and their working concentration were listed in Supplementary Table 2. The NF-κB inhibitor PDTC (S1808) and ROS assay kit (S0033) were purchased from Beyotime Institute of Biotechnology (Nantong, Jiangsu). MitoTEMPO (SML0737) was purchased from Sigma-Aldrich (St Louis, MO). TLR9 antagonist (ODN INH-18) was purchased from InvivoGen (San Diego, CA). DNase I (4393898), MitoTracker Deep Red FM (Molecular Probes, M22425), and PicoGreen (Molecular Probes, P7581) were purchased from Invitrogen (Carlsbad, CA). PULSin protein delivery reagent was purchased from Polyplus transfection Inc. (New York, NY). Human CCL2/MCP-1 (DCP00), CCL17/TARC (DDN00), CCL22/MDC (DMD00), and IL-10 (D1000B) Quantikine ELISA Kit were purchased from R&D Systems (Minneapolis, MN).

### Animals

Total 20 six-week-old male BALB/c nude mice were randomly divided into five groups. In this study, we did not exclude animals, as the mice did not observe any abnormality in weight or apparent disease symptoms before performing experiments. Orthotopic HCC model was induced by established by orthotopically introhepatic injection of HCC cell as previously described [9]. In antagonist treatment group, mice were intraperitoneally injected with 50 mg/kg CCR2 antagonist (sc-202525, Santa Cruz Biotechnology, Santa Cruz, CA) by every other day after tumor cell implantation. One week after tumor implantation, 5 × 10^5^ THP-1 macrophages in 0.1 ml RPMI-1640 were injected into the portal vein weekly in macrophage treatment groups. All mice were sacrificed 5 weeks after intrahepatic implantation. The weight of tumor was measured, and tumor tissue samples were evaluated by hematoxylin and eosin (H&E) staining and immunohistochemistry. Animal study was approved by the ethics committee of the FMMU.

### qPCR, western blot, IHC, and H&E staining

Total RNA extraction, complementary DNA synthesis, and qPCR were performed as previously described [8]. Primer sequences of qPCR were provided in Supplementary Table 3. IHC staining was performed as previously described using Histostain-Plus Kit (Invitrogen, Carlsbad, CA) [42]. The percentage of positively stained macrophages to the total number of visible cells in each sample was determined using computerized analysis. H&E staining was carried out following standard procedures. Western blot analysis of cell lines was performed as previously described [42].

### Confocal microscopy, immunofluorescence labeling, and flow cytometry

MitoTracker Deep Red FM was used to monitor mitochondrial morphology according to the manufacturer’s instructions using Olympus FV 1000 laser-scanning confocal microscope (Olympus Corporation, Tokyo). For morphometric analysis, Image J software (NIH, Bethesda, MD) was used to measure the length of mitochondria. The distribution of mitochondrial nucleoids in living HCC cells was stained by Picogreen according to the manufacturer’s instructions. To confirm the co-localization of mtDNA and TLR9, the HCC cells were first fixed with 4% paraformaldehyde in phosphate-buffered saline (PBS; pH 7.4) and permeabilized with 0.1% Triton X-100, and then incubated with PicoGreen and PE-labeled TLR9 antibody. For immunofluorescence labeling, THP-1 macrophages were incubated with CM for 48 h and washing three times with RPMI-1640 and then incubated with PicoGreen and PE-conjugated CD163 antibody for another 1.5 h. Cells were immediately washed three times with PBS and observed with confocal microscope.

For flow cytometry, THP-1 macrophages were collected after incubation with different CM for 48 h. The cells were washed three times with cold PBS containing 5% human serum and 0.1% NaN_3_ and then incubated with the PE (Phycoerythrin)-conjugated CD163 antibody for 1 h. Immediately, cells were analyzed by Cytomics FC500 (Beckman Coulter, Fullerton, CA).

Flow cytometry analysis was performed to detect the intracellular ROS by 2’,7’-dichlorofluorescein diacetate (DCFH-DA) fluorescent probe using ROS assay kit as previously described [8].

### Determination of relative mtDNA copies in cytosolic extracts

Cytosol was isolated using the cell mitochondria isolation kit (C3601, Beyotime Institute of Biotechnology, Nantong, Jiangsu) according to the manufacturer’s instructions as previously described [37, 43]. In brief, 1 × 10^7^ cells were incubated in 0.1 ml ice-cold mitochondrial lyses buffer for 10 min and homogenized with a Dounce homogenizer for 30 strokes. The homogenate was centrifuged at 600 × *g* for 10 min at 4 °C to remove nuclei and unbroken cells. The supernatant was collected and centrifuged again at 12,000 × *g* for 30 min at 4 °C for production of a supernatant corresponding to the cytosolic fraction. DNA of cytosolic fractions were isolated using QIAQuick nucleotide removal kit (28306, QIAGEN, Valencia, CA) following the manufacturer’s protocol. The copy number of mtDNA was measured by qPCR with same volume of the DNA solution as previously described [44].

### Migration assay

Twenty-four-well transwell plates (Corning Inc., New York, NY) were used to examine the migration of macrophages induced by CM from HCC cells with different treatments. THP-1 macrophages were collected and added into the top chamber of 24-well transwell plates. Simultaneously, CM and RPMI-1640 medium containing 20% FBS were added into the bottom of transwell chamber. After 24 h, the cells that crossed the inserts were stained with crystal violet and counted under phase-contrast microscopy. Five fields were randomly selected and the average number of inserted cells was calculated.

### DNase I treatment

Cells were seeded at 5 × 10^4^ cells/well in 24-well plates and cultured for 24 h. Before transfection, PULSin/DNaseI mixture was prepared according to the manufacturer’s instructions. Then, cells were washed three times using serum-free RPMI-1640 and transfected with 3 μg of DNase I using PULSin™ reagent for 4 h at 37 °C. After removing the media, cells were incubated in fresh complete medium for 24 h and the CM and cells were collected for further studies.

### Enzyme-linked immunosorbent assay

To measure CCL2 concentration, HCC cells were incubated in a serum-free medium for 48 h after different treatments and the culture supernatant was harvested for further assay. To measure IL-10, CCL17, and CCL22 concentration, THP-1 macrophages were incubated with CM for 48 h. After washing three times with PBS, the cells were incubated in serum-free medium for 48 h and the culture supernatant was harvested for further assay. The concentration of CCL2, IL-10, CCL17, and CCL22 was measured with ELISA Kit following the manufacture’s protocol.

### Statistical analysis

All experiments were technically repeated three times, where appropriate. SPSS 19.0 software (SPSS, Chicago, IL) was used for all statistical analyses and *p* < 0.05 was considered statistically significant. Unpaired Student’s *t*-tests (two-sided) were used for comparisons between two groups where appropriate. Error bars represent standard error of mean. Correlations between measured variables were tested by Spearman rank correlation analyses. For prognosis analysis, variables (the IHC score of Drp1, CCL2, TLR9, and the percentage of CD163^+^ cells) were analyzed dichotomically. The Kaplan-Meier survival curve and log-rank test were used to distinguish subgroup patients who had different overall survival. People who performed lab work were blinded to patients’ clinical data and no blinding was done for all animal studies. For every figure, the statistical tests are justified as appropriate and the data meet the assumptions of the tests.

## Supplementary information

Supplementary Figure.5.

Supplementary Figure.1.

Supplementary Figure.2.

Supplementary Figure.3.

Supplementary Figure.4.

Supplementary Info Clean.
