# Supplementary figures and images for: Mitochondrial fission-induced mtDNA stress promotes tumor-associated macrophage infiltration and HCC progression
Source: Oncogene. 2019 Mar 20;38(25):5007–20. doi: 10.1038/s41388-019-0772-z (PMC6755992; doi:10.1038/s41388-019-0772-z)

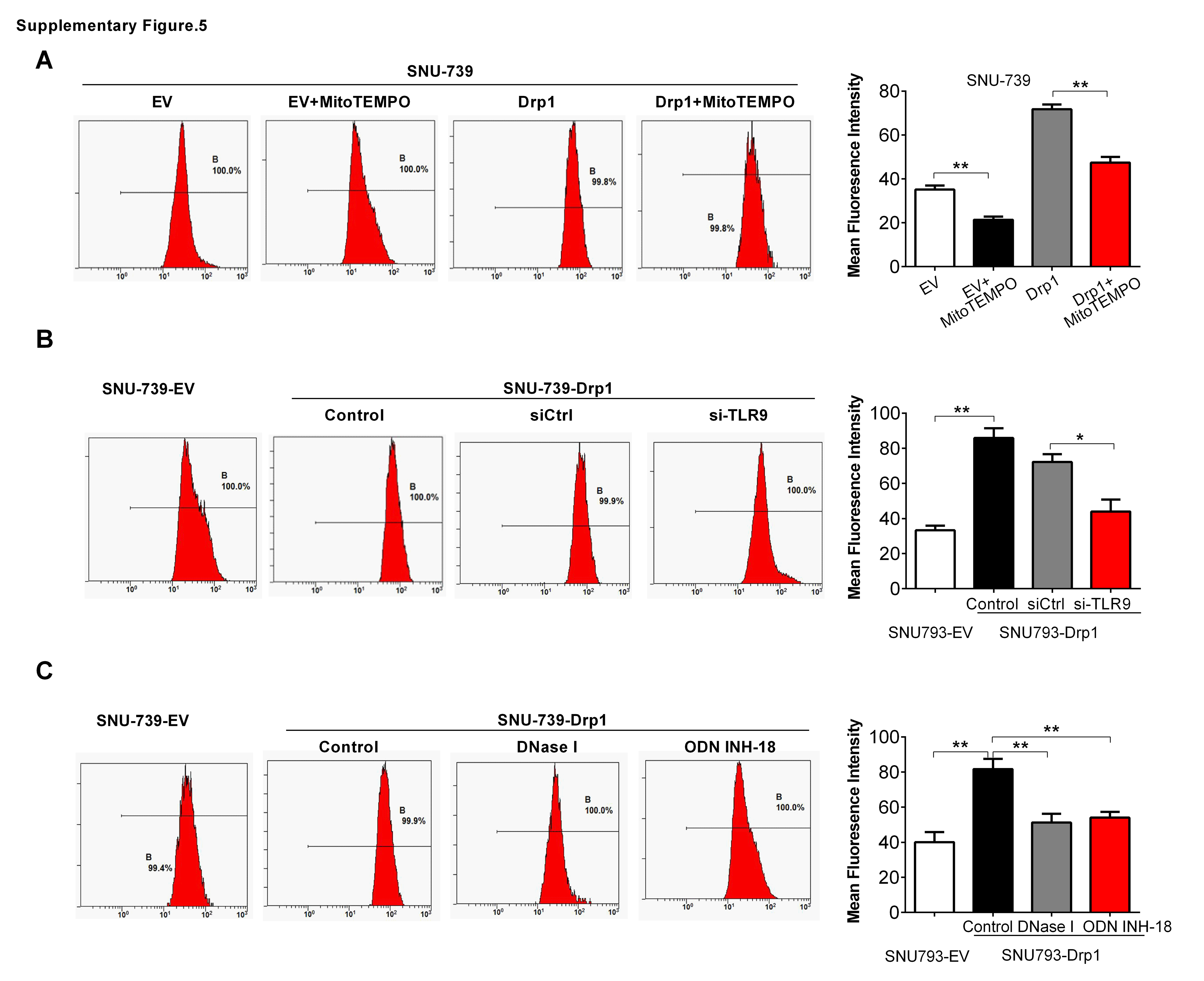

Supplement: Supplementary file 1 — Supplementary Figure.5. [file 41388_2019_772_MOESM1_ESM.tif]

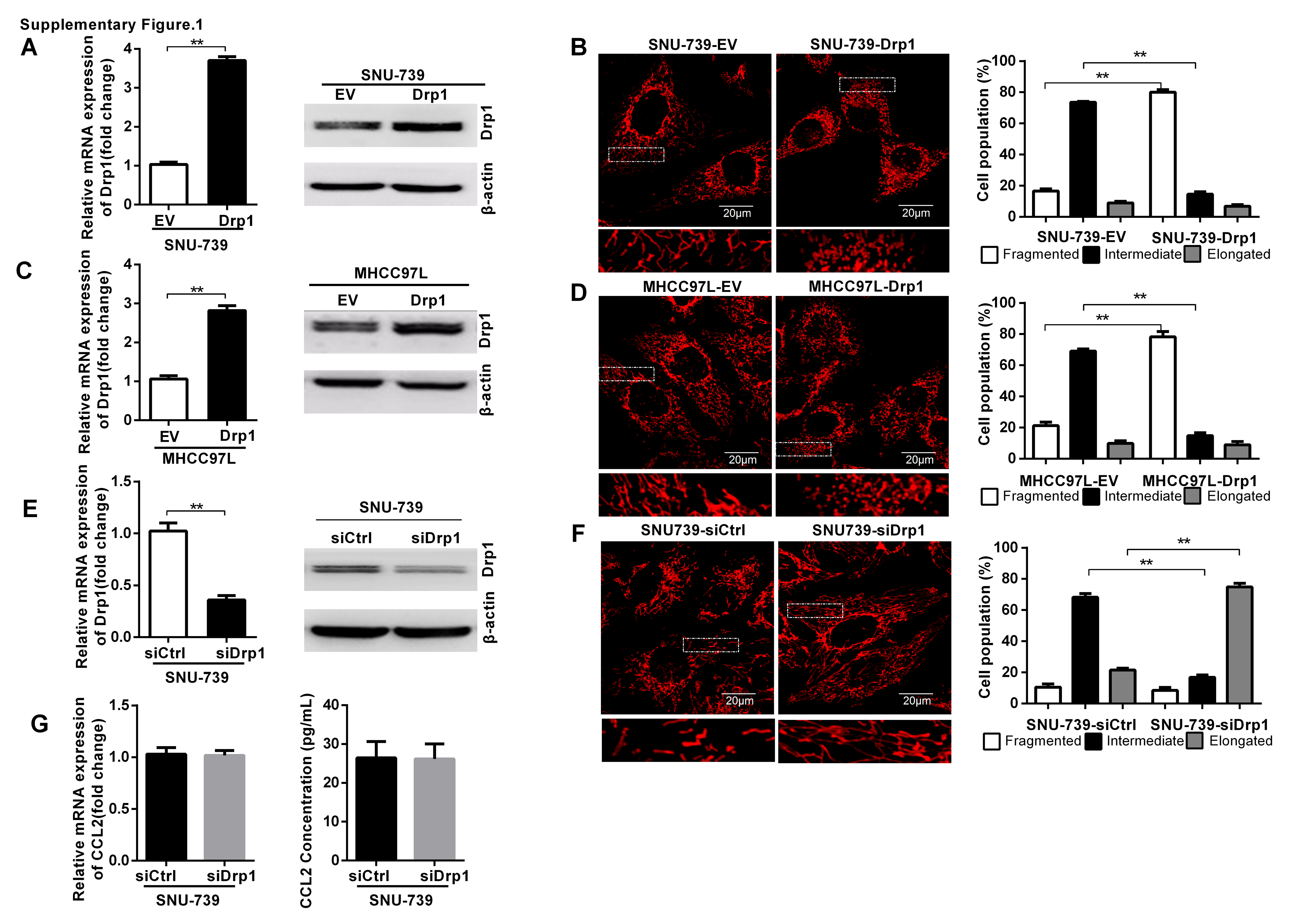

Supplement: Supplementary file 2 — Supplementary Figure.1. [file 41388_2019_772_MOESM2_ESM.tif]

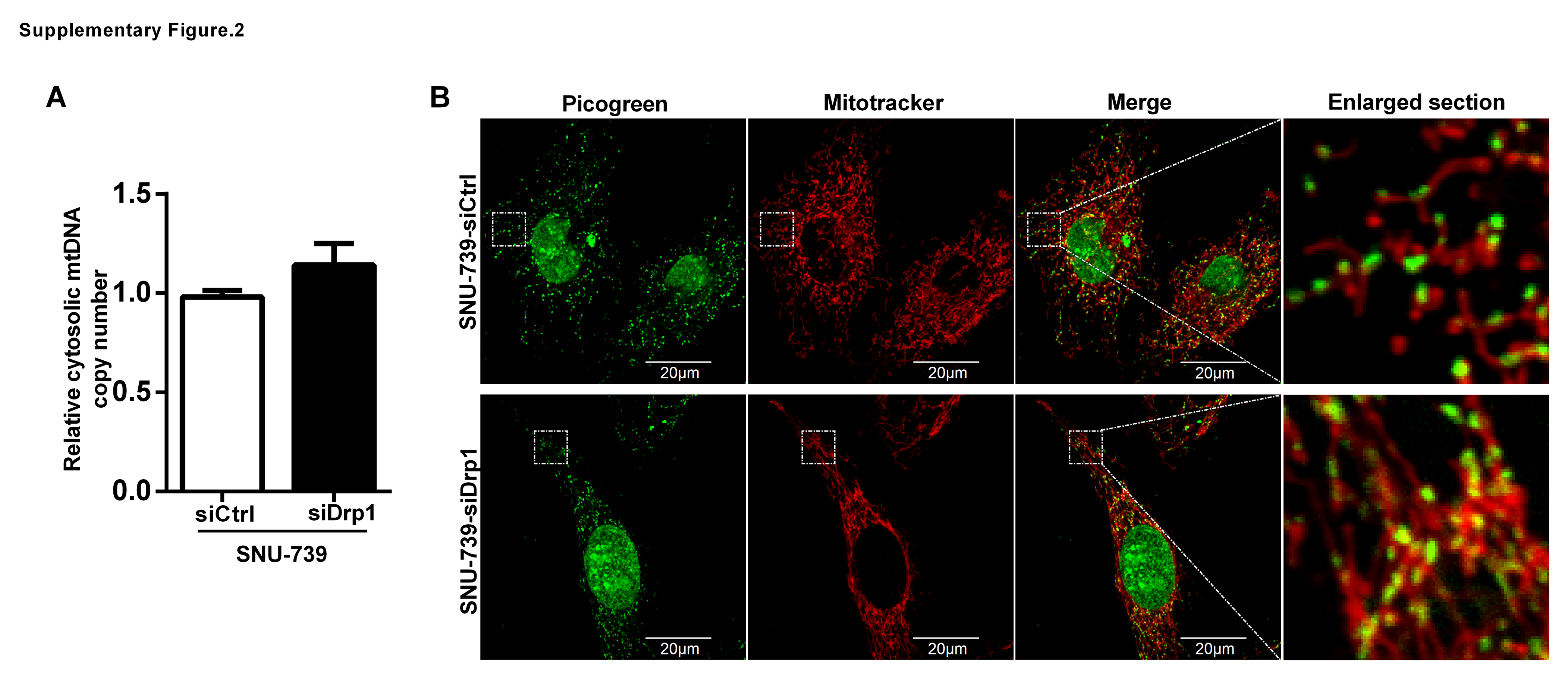

Supplement: Supplementary file 3 — Supplementary Figure.2. [file 41388_2019_772_MOESM3_ESM.tif]

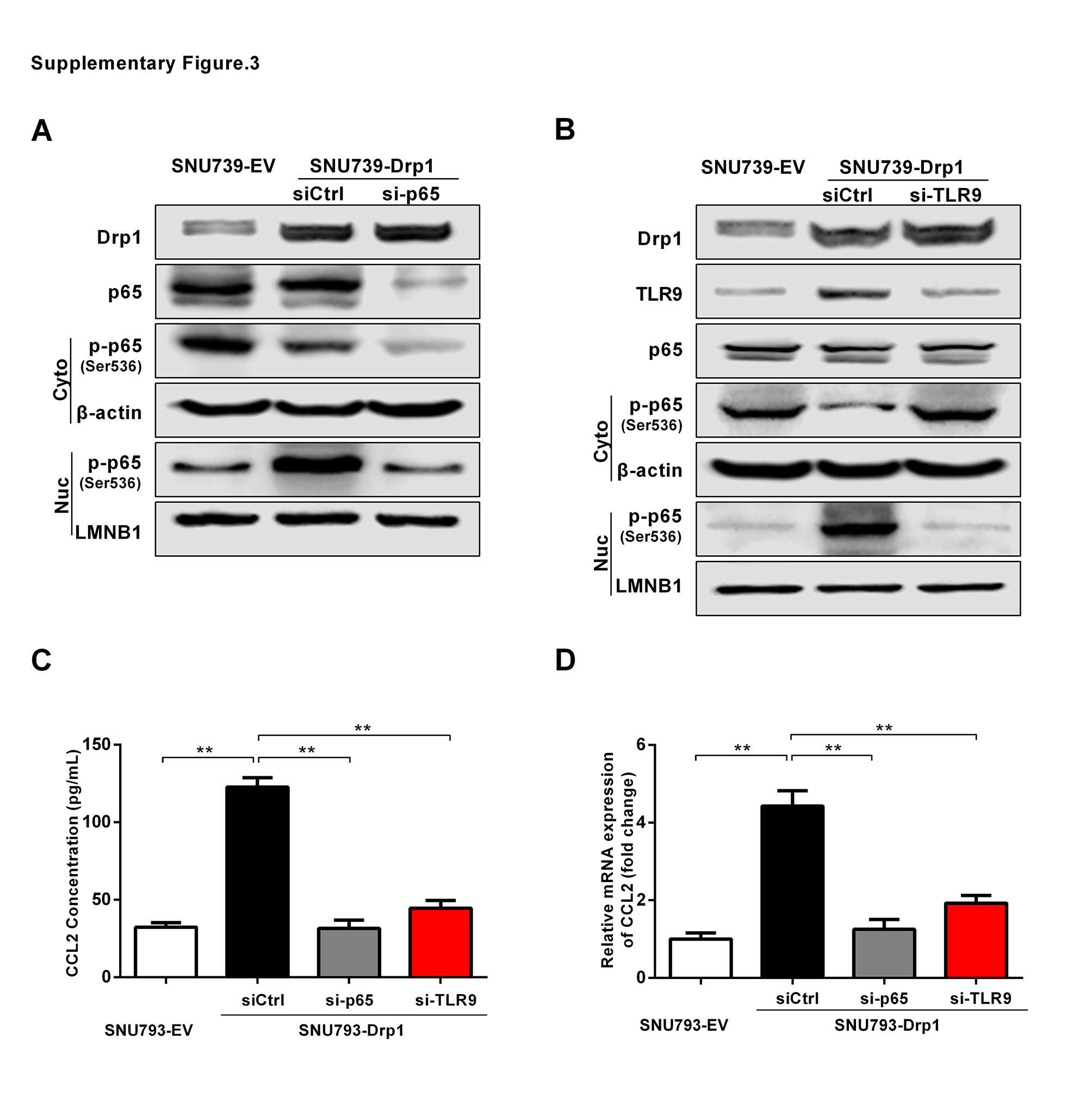

Supplement: Supplementary file 4 — Supplementary Figure.3. [file 41388_2019_772_MOESM4_ESM.tif]

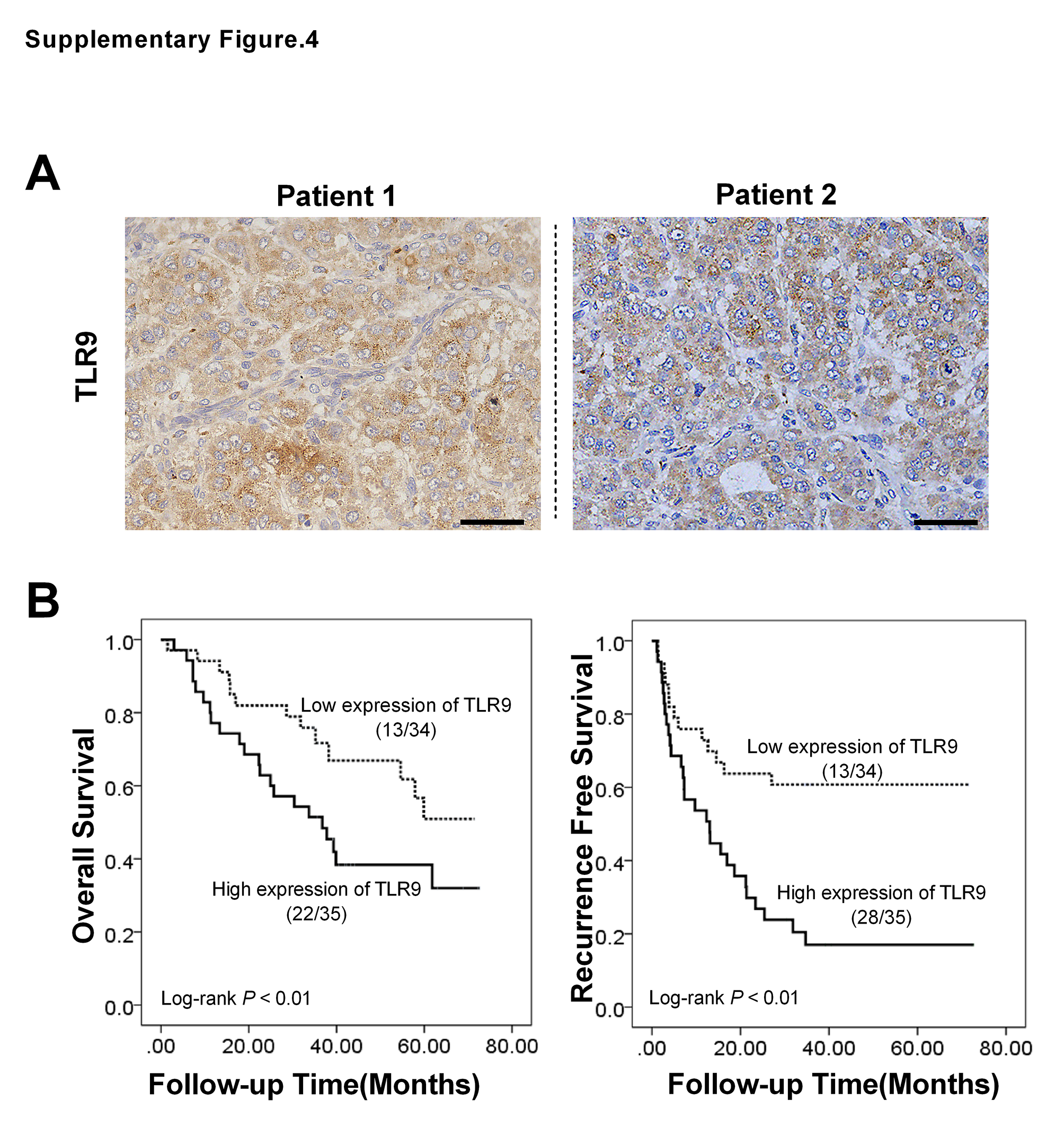

Supplement: Supplementary file 5 — Supplementary Figure.4. [file 41388_2019_772_MOESM5_ESM.tif]
