# Supplementary material for: Mitochondrial fission-induced mtDNA stress promotes tumor-associated macrophage infiltration and HCC progression
Source: Oncogene. 2019 Mar 20;38(25):5007–20. doi: 10.1038/s41388-019-0772-z (PMC6755992; doi:10.1038/s41388-019-0772-z)
Supplement: Supplementary file 6 — Supplementary Info Clean. [file 41388_2019_772_MOESM6_ESM.docx]

**Supplementary information**

**Mitochondrial fission****-induced mtDNA stress promotes tumor-associated macrophage infiltration and HCC progression**

Dengke Bao^1,2,*^; Jing Zhao^1,2,*^; Xingchun Zhou^2,*^; Qi Yang^1^; Yibing Chen^3^; Jianjun Zhu^2^; Peng Yuan^2^; Jin Yang^4^, Tao Qin^5^, Shaogui Wan^1,#^; Jinliang Xing^2,#^

**Supplementary Table**

Supplementary Table 1. Distribution of HCC patients’ characteristics

| **Variable** | **All patients, n (%), n = 69** |
| --- | --- |
| Sex |  |
| Female | 8 (11.6%) |
| Male | 61 (88.4%) |
| Age, years |  |
| <52 | 38 (55.1%) |
| ≥52 | 31 (44.9%) |
| Serum HBsAg |  |
| Negative | 3 (4.3%) |
| Positive | 66 (95.7%) |
| Serum AFP, ng/ml |  |
| <200 | 37 (53.6%) |
| ≥200 | 32 (46.4%) |
| Size of tumor, cm |  |
| <5cm | 32 (46.4%) |
| ≥5cm | 37 (53.6%) |
| PVT |  |
| No | 63 (91.3%) |
| Yes | 6 (8.7%) |
| TNM stage |  |
| I+II | 26 (37.7%) |
| III+IV | 43 (62.3%) |
| Survival |  |
| Death | 35 (50.7%) |
| Alive | 34 (49.3%) |

PVT, portal vein thrombus; TNM, tumor node metastasis; AFP, alphafetoprotein.

Supplementary Table 2. Antibodies and their working concentration used in this study.

| **Antibody** | **Company (Cat. No.)** | **Working dilutions** |
| --- | --- | --- |
| Drp1 | abcam (ab56788) | WB: 1/800;  IHC: 1/200 |
| CCL2 | - Sigma-Aldrich (HPA019163) | IHC: 1/200 |
| CD163 | Thermo Fisher Scientific (MA5-11458) | IHC: 1/100 |
| TLR9 | Thermo Fisher Scientific (PA5-27258) | IHC: 1/200  WB:1/500 |
| NF-κB p65 (D14E12) | Cell Signaling (8242) | WB:1/500 |
| Phospho-NF-κB p65 (Ser536) | Cell Signaling (13346) | WB:1/500 |
| Actin | Beijing TDY BIOTEC CO., Ltd. (TDY051C) | WB: 1/3000 |
| LMNB1 | Beijing TDY BIOTEC CO., Ltd. (TDY049) | WB: 1/2000 |
| PE Mouse Anti-Human CD163 | BD Pharmingen (556018) | Confocal Microscopy: 1:50; Flow cytometry: 20µl/Test |
| PE Rat Anti-Human TLR9 | BD Pharmingen (560425) | Confocal Microscopy: 1:50 |
| Goat anti-Rabbit IgG (H+L), HRP Conjugated | Beijing TDY BIOTEC CO., Ltd. (S-004F) | WB: 1:8000 |
| Goat anti-Mouse IgG (H+L), HRP Conjugated | Beijing TDY BIOTEC CO., Ltd. (S-001F) | WB: 1:8000 |

Supplementary Table 3. Sequence of primers for qRT-PCR and siRNA.

|  | **Primers for qRT-PCR** |  |
| --- | --- | --- |
| **Target** | **Forward primer (5’-3’)** | **Reverse primer (5’-3’)** |
| Drp1 | GGAGACTCATCTTTGGTGAAGAG | AAGGAGCCAGTCAAATTATTGC |
| CCL2 | CAGCCAGATGCAATCAATGCC | TGGAATCCTGAACCCACTTCT |
| CCL17 | ATGGCCCCACTGAAGATGCT | TTGGGGTCCGAACAGATGG |
| IL-4 | GACATCTTTGCTGCCTC | TACTCTGGTTGGCTTCCTTCA |
| IL-13 | ATCCTCTCCTGTTGGCAC | CTGGTTCTGGGTGATGTTGAC |
| TGF-β | GGCGATACCTCAGCAACCG | CTAAGGCGAAAGCCCTCAAT |
| IL-10 | TCCCTGTCAAAACAAGAGCA | ATAGAGTCGCCACCCTGATG |
| CCL22 | TGATTACGTCCGTTACCGTCT | CCTGAAGGTTAGCAACACCAC |
| VEGF | TGGGTGCATTGGAGCCTTGCCTTGC | GGCAGTAGCTGCGCTGATAGACATCC |
| TLR9 | CCGTGACAATTACCTGGCCTTC | CAGGGCCTTCAGCTGGTTTC |
| CD68 | GCTACATGGCGGTGGAGTACAA | ATGATGAGAGGCAGCAAGATGG |
| CD163 | TTTTGTCACCAGTTCTCTTGGA | AGCCATTATTACACACGTTCC |
| GAPDH | AACGGATTTGGTCGTATTGG | TTGATTTTGGAGGGATCTCG |
| ND1 | CCCTAAAACCCGCCACATCT | GAGCGATGGTGAGAGCTAAGGT |
| HGB1 | GTGCACCTGACTCCTGAGGAGA | CCTTGATACCAACCTGCCCAG |
|  | **Sequence of siRNA** |  |
| **Target** | **Sense (5’-3’)** | **Anti-sense (5’-3’)** |
| Drp1 | ACUAUUGAAGGAACUGCAAAAUAUATT | UAUAUUUUGCAGUUCCUUCAAUAGUTT |
| p65 | CAGAUACAGACGAUCGUCATT | UGACGAUCGUCUGUAUCUGTT |
| TLR9 | GCCUUUCCUUGUCCUCCAATT | UUGGAGGACAAGGAAAGGCTT |
| Control | UUCUCCGAACGUGUCACGUTT | ACGUGACACGUUCGGAGAATT |

**Supplementary figure**

**Supplementary Figure. 1**


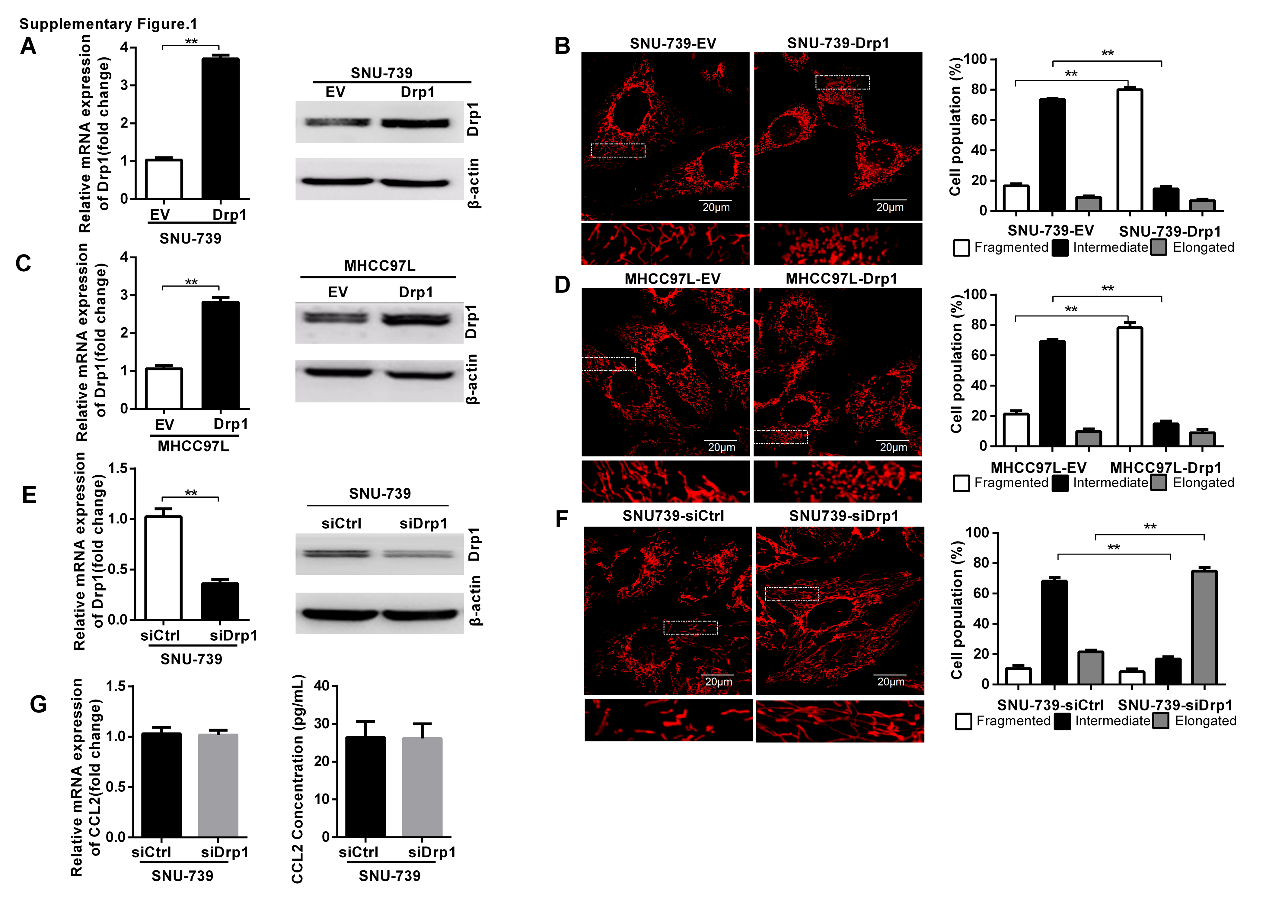


(A), (C) and (E) qRT-PCR and western blot analysis for the mRNA expression and protein levels of Drp1 in HCC cells as indicated. (B), (D) and (F) Confocal microscopy analysis of mitochondrial network in different HCC cells as indicated with MitoTracker (Mito.). The proportion of HCC cells (n=100 cells for each sample) with fragmented, intermediate, and elongated mitochondria was quantified. (G) qRT-PCR analysis for the mRNA expression levels and ELISA assay for the secretion of CCL2 in HCC cells as indicated. EV indicated as cells transfected with empty vector; Drp1 indicated as cells transfected with expression vector encoding Drp1. siCtrl indicated as cells transfected with control siRNA; siDrp1 indicated as cells transfected with siRNAs against Drp1. **, *P* < 0.01.

**Supplementary Figure. 2**


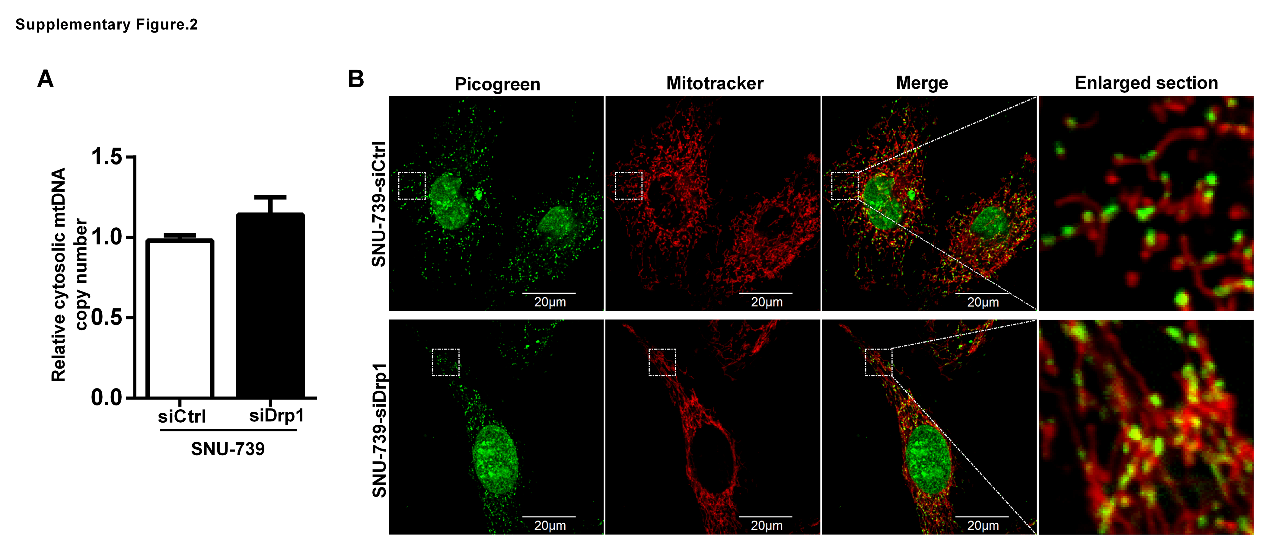


(A) Cytosolic mtDNA copy number was quantitated via qPCR in HCC cells as indicated. HGB1 was used as internal control. (B) Confocal microscopy images of indicated HCC cells stained with Picogreen (DNA) and MitoTracker (Mito.). siCtrl indicated as cells transfected with control siRNA; siDrp1 indicated as cells transfected with siRNAs against Drp1.

**Supplementary Figure. 3**


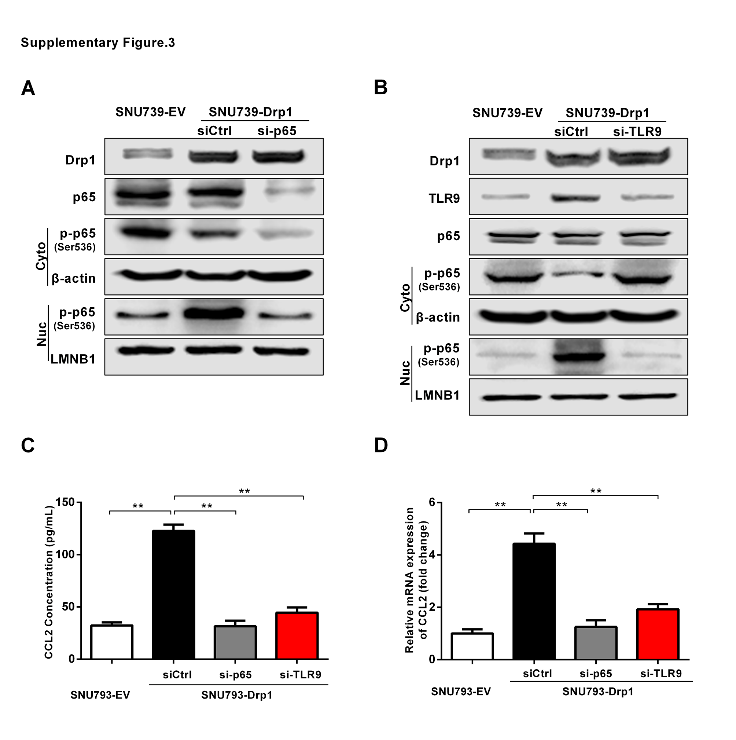


(A) and (B) Western blot analyses for protein levels of Drp1 and NF-κB activation-related molecules in whole-cells or p65 in cytoplasm and nucleus of HCC cells as indicated treatment. (C) ELISA analysis of the CCL2 concentration in the supernatants of cultured HCC cells with treatment as indicated. (D) qRT-PCR analysis for the mRNA expression levels of CCL2 in HCC cells as indicated. EV indicated as cells transfected with empty vector; Drp1 indicated as cells transfected with vector expressing Drp1. siCtrl indicated as cells transfected with control siRNA; si-p65 indicated as cells transfected with siRNAs against p65; si-TLR9 indicated as cells transfected with siRNAs against TLR9; p65, NF-κB p65; p-p65, phosphorylated NF-κB p65. **, P < 0.01.

**Supplementary Figure. 4**


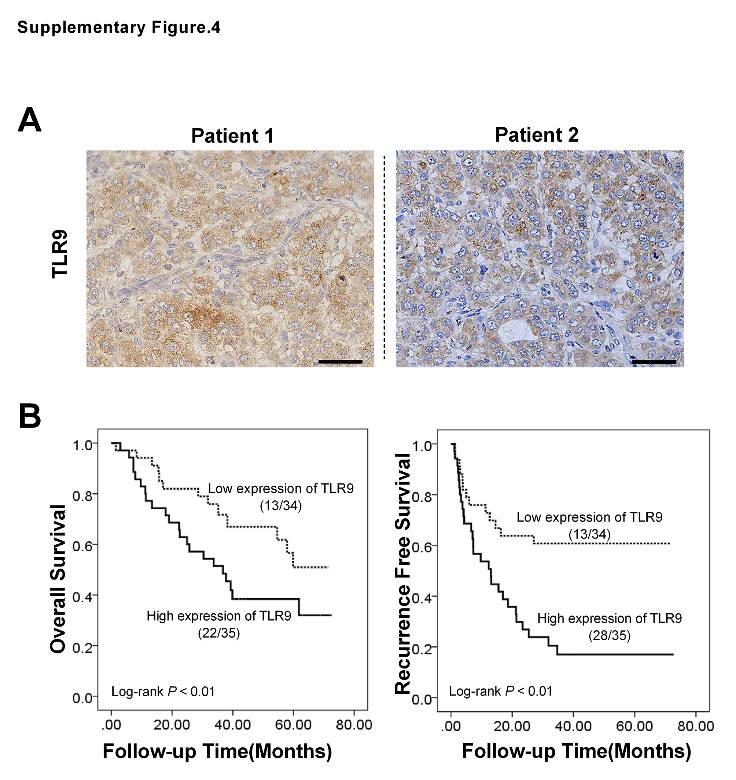


(A) Representative immunohistochemical (IHC) staining images of TLR9 in representative HCC tissues (n = 69). Scale bar: 50μm. (B) Kaplan-Meier curve analysis of overall survival and recurrence free survival in HCC patients by the expression of TLR9 in HCC tissues. Patients were divided into high or low level by the median value of TLR9 expression for further analysis. Death/total and recurrence/total number of patients in each subgroup were presented. **, *P* < 0.01.

**Supplementary Figure. 5**


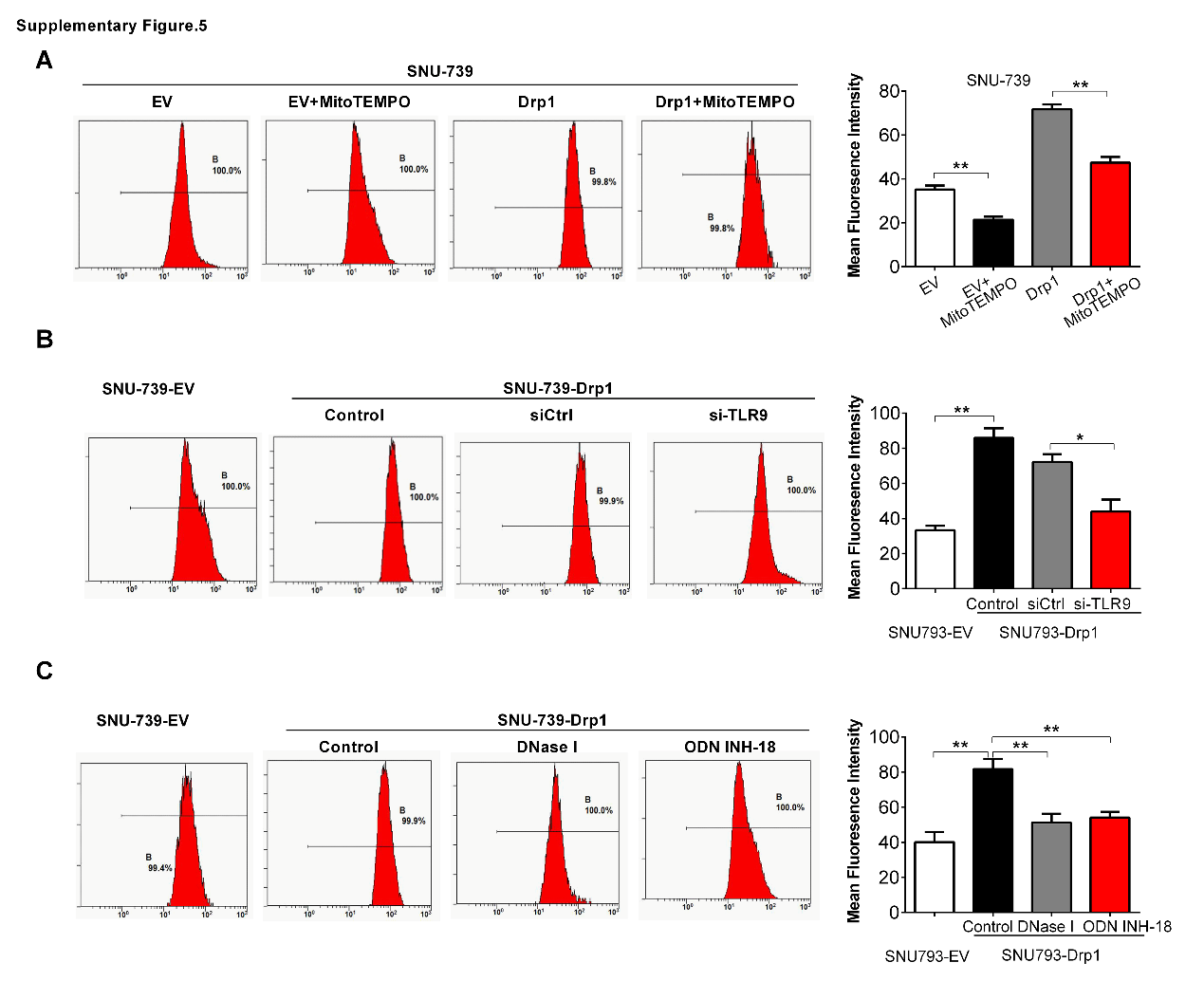


(A), (B) and (C) Intracellular ROS levels were analyzed by flow cytometry in HCC cells treated as indicated, and the results were expressed as mean fluorescence intensity. EV indicated as cells transfected with empty vector; Drp1 indicated as cells transfected with vector expressing Drp1. siCtrl indicated as cells transfected with control siRNA; si-TLR9 indicated as cells transfected with siRNAs against TLR9; DNase I; HCC cells treated with DNase I preparations; ODN INH-18, HCC cells treated with TLR9 antagonist (ODN INH-18); MitoTEMPO, HCC cells treated with scavenger of mitochondrial superoxide; p65, NF-κB p65; p-p65, phosphorylated NF-κB p65. *, P < 0.05; **, P < 0.01.
